# Memristor Neural Network Training with Clock Synchronous Neuromorphic System

**DOI:** 10.3390/mi10060384

**Published:** 2019-06-08

**Authors:** Sumin Jo, Wookyung Sun, Bokyung Kim, Sunhee Kim, Junhee Park, Hyungsoon Shin

**Affiliations:** 1 Department of Electronic and Electrical Engineering, Ewha Womans University, Seoul 03760, Korea; sumin5784@gmail.com (S.J.); wkyungsun@ewha.ac.kr (W.S.); bkkim0505@ewhain.net (B.K.); 2 Department of System Semiconductor Engineering, Sangmyung University, Cheonan 31066, Korea

**Keywords:** neuromorphic system, Hebbian training, guide training, memristor, image classification

## Abstract

Memristor devices are considered to have the potential to implement unsupervised learning, especially spike timing-dependent plasticity (STDP), in the field of neuromorphic hardware research. In this study, a neuromorphic hardware system for multilayer unsupervised learning was designed, and unsupervised learning was performed with a memristor neural network. We showed that the nonlinear characteristic memristor neural network can be trained by unsupervised learning only with the correlation between inputs and outputs. Moreover, a method to train nonlinear memristor devices in a supervised manner, named guide training, was devised. Memristor devices have a nonlinear characteristic, which makes implementing machine learning algorithms, such as backpropagation, difficult. The guide-training algorithm devised in this paper updates the synaptic weights by only using the correlations between inputs and outputs, and therefore, neither complex mathematical formulas nor computations are required during the training. Thus, it is considered appropriate to train a nonlinear memristor neural network. All training and inference simulations were performed using the designed neuromorphic hardware system. With the system and memristor neural network, the image classification was successfully done using both the Hebbian unsupervised training and guide supervised training methods.

## 1. Introduction

Neuromorphic hardware research has begun to develop new computing architectures [1,2,3,4,5,6]. From a broad point of view, neuromorphic research has two main streams [6]. One focuses on reproducing the exact biological phenomena that occur in the brain [3,6,7,8,9,10], while the other focuses on the development of a new computing device typically known as a neuromorphic chip. As the neuromorphic chip takes advantage of the biological neural network, it has several features such as massively parallel processing, local memory structure, high integrity, and low power consumption [4,5,11,12,13,14,15,16,17,18,19,20,21,22]. Neuromorphic hardware is especially efficient in terms of size and power consumption compared to typical Von Neumann architecture computing devices. The main difference between neuromorphic hardware and Von Neumann computers is the memory structure. In the human brain, the neural cell topology is determined by the connections between neurons (i.e., synaptic connectivity). This means that the biological neural network contains a memory device and a computing unit at the same time. On the contrary, the memory device and computing unit are separated in a typical Von Neumann computer. Most of the power is consumed from the data transfer between the memory device and computing unit. This power issue appears in extreme forms in recent data-intensive artificial intelligence (AI) applications. 

There are approximately 100–500 trillion synapses in the adult human brain [23]. A memristor can be very densely integrated but remain energy efficient. Therefore, it has considerable potential to physically implement huge and complex network connectivity similar to the human brain [8,24,25,26,27]. In addition to this integration property, its I–V characteristic makes the memristor device an appropriate synapse device. It was first suggested and reported that the I–V characteristic is analogous to the behavior of biological synapses in [28]. Due to this device characteristic, memristors have been considered to have the potential to implement spike timing-dependent plasticity (STDP) in hardware. Neural networks can learn by themselves based on given information (i.e., unsupervised learning). STDP is one of the types of unsupervised learning methods in the brain. This concept is in contrast to supervised learning, which is learning processed as the supervisor intended. Supervised learning needs prior information about processing data, and the supervisor needs to label all the data. As the amount of data to process has increased, this labeling process has become more demanding. Natural data is continuously changing, and it is difficult to label all the input data. Thus, unsupervised learning is more appropriate to deal with natural data than supervised learning.

Unsupervised learning has a simpler mechanism than supervised learning. Training a multilayer artificial neural network (ANN), however, requires accurate data control over the entire network (i.e., input/output of the network and input/output of the layers in the network). The systematic implementation of unsupervised learning in a multilayer ANN is essential to develop neuromorphic hardware whose basic function is analogous to the biological neural network, and that can consequently process natural data. From the user-centered point of view, however, with unsupervised learning, it is difficult to determine whether the training has been completed, and the accuracy can be lower than that of supervised learning. On the other hand, users can train the ANN as they intend with supervised learning, it is easier to analyze the training results, and there are many methods to improve accuracy. However, the machine learning algorithms used to train ANNs need computations based on current synaptic weights. In addition to the computations, those computed synaptic weights have to be applied exactly and updated. Extra effort is needed to measure the resistance of a single memristor device in the memristor neural network and to record the entire hysteresis. Only then can the memristor resistance be accurately modified. These accompanied processes compromise the energy efficiency and integrity of memristor neural networks. Therefore, it is hard to realize supervised learning when the ANN consists of a memristor device. 

Considering the circumstances of neuromorphic hardware implementation with a memristor ANN, a clock synchronous neuromorphic hardware system for both supervised learning and unsupervised learning was designed in this paper. The designed system was available for a multilayer memristor ANN with an unsupervised learning method. A guide-training algorithm capable of training a nonlinear memristor neural network in a supervised manner without backpropagation was devised in addition to the neuromorphic system. A memristor ANN was adopted as the synapse array for the designed neuromorphic hardware system, and the memristor ANN was trained in both unsupervised learning with the Hebbian training algorithm and supervised learning with the guide-training algorithm. 

## 2. Materials and Methods

### 2.1. Clock Synchronous Neuromorphic Hardware System

The control of network input/output and neural layer input/output is the most important aspect of implementing unsupervised learning in an ANN. In supervised learning, the network input is completely processed through the network, and then network output is computed. The synaptic weights are then updated according to the backpropagation. On the contrary, unsupervised learning, such as the Hebbian or STDP algorithm, updates the synaptic weights only based on the correlations between inputs and outputs. There is a single pair of input and output in a single-layer ANN, and the correlation between them is clear. However, the situation changes when it comes to the multilayer ANN. Based on the network structure, the layers can be wide or deep, and the number of neurons contained in each layer differs. As a result, the data processing time between layers also differs. For instance, consider the circumstances in a 9-6-3 double-layer ANN (nine input neurons and six output neurons in layer 1 (L1) and six input neurons and three output neurons in layer 2 (L2)). The input to L1 is the network input (NI), and the output of L2 is the corresponding network output (NO). The output of L1 (L1out) is input to L2 (L2input). First, the network input NI1 is applied and the corresponding L1 output (L1out1) is propagated to L2, but the corresponding L2 output (NO1) is not computed yet. What happens if the second network input NI2 is applied again? In the best case, the corresponding L2 output for L2iniput1 is computed, and then the L1 output corresponding to the NI2 (L1out2) is applied to L2. However, in the worst case, the L1 output corresponding to the NI2 (L1out2) is applied to L2 before L2input1 is computed. As a result, the synaptic weights of layer 1 are updated based on the correlations between two different inputs NI1, NI2, and two different outputs L1out1 and L1out2. However, the synaptic weights of layer 2 are updated based on the correlations between two different inputs L2input1, L2input2 and a single merged output of two different inputs. This kind of timing error can result in a learning error, and there are far more possibilities in a deeper ANN than this example case. 

**Figure 1:**
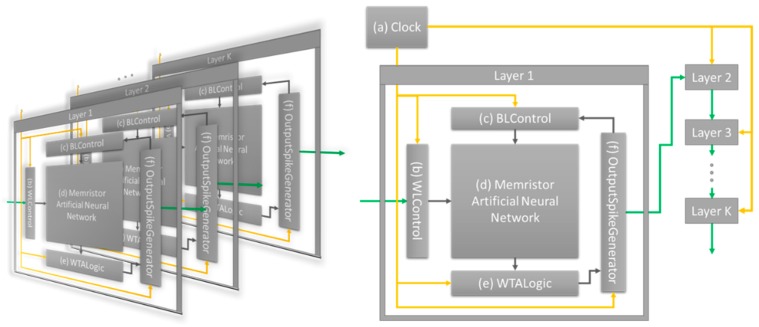
Clock synchronous neuromorphic hardware system. (**a**) System clock; (**b**) Word line control logic; (**c**) Bit line control logic; (**d**) memristor artificial neural network; (**e**) winner-takes-all logic; and (**f**) output spike-generating logic.

The neuromorphic system proposed in this paper divides the data process to a single input data into four steps, and synchronizes the entire layer with the clock: allowing the input to be received by the layer, computing the output, updating the synaptic weights, propagating the output. This system can perform unsupervised learning without timing error. In accordance with the clock signal (Clock, Figure 1a), the four processing steps are performed by word line control logic (WLControl, Figure 1b), bit line control logic (BLControl, Figure 1c), output computation block (WTALogic, Figure 1e), and output propagating logic (OutputSpikeGenerator, Figure 1f). All layers of the ANN simultaneously receive the data, compute the output, update the synaptic weights based on the input and output, and propagate the output to the next layer. At this point, the propagated output from the previous layer is not instantly applied to the next layer. Rather, it is applied to the layer as the next clock signal for receiving input data. The aforementioned timing error can be improved with this neuromorphic hardware system. All training and inference simulations are performed using this designed system. The memristor ANN (Figure 1d) is applied to the neuromorphic hardware system, and both Hebbian training and guide training are performed and analyzed. The detailed methods of Hebbian training and guide training are explained in Section 2.3 and Section 2.4, respectively, and the corresponding training and inference results are presented in Section 3.1 and Section 3.2, respectively. 

### 2.2. Memristor Neural Network Array

**Figure 2:**
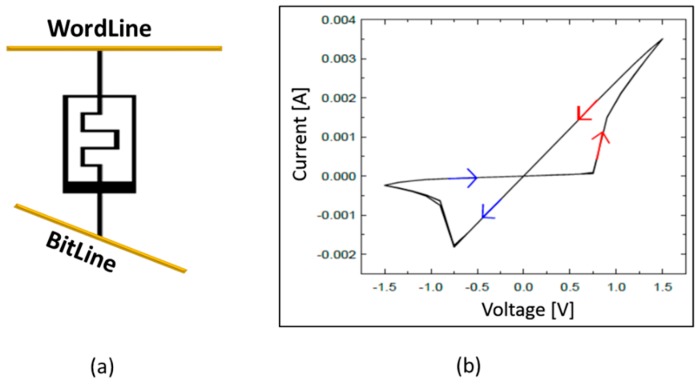
(**a**) Memristor neural network structure; and (**b**) I–V characteristic of memristor device model used in this paper.

As shown in Figure 2a, the memristor device is connected between the top electrode (Word Line, WL) and the bottom electrode (Bit Line, BL). Input data were applied as voltage to the WL, and the current flowed through the memristor from the WL to the BL according to the input voltage. The winner-takes-all logic (Figure 1e) determined the neuron where the largest current flows. Based on this computation, OutputSpikeGenerator (Figure 1f) propagated output spikes to BLControl (Figure 1c) and the next layer. WLControl and BLControl apply the appropriate voltage to modify memristor conductance according to the learning algorithm. 

Memristor devices can be modeled using various parameters in the equations, and various models have been reported [29,30,31,32,33]. The memristor device model used in this study refers to References [31] and [32]. The simulation result in Figure 6 of reference [32] is based on the experimental data in [33]. In this study, we used a memristor neural network and a peripheral neuromorphic system instead of a single memristor device. Therefore, the modeling parameters were adjusted to the 1.5 V of system operating voltage while maintaining the device current analogous to the experimental data in [33]. The modeling parameters used are shown in Table 1. The I–V characteristic of the memristor device model used in this paper is shown in Figure 2b. To change the memristor device resistance, a voltage larger than 0.75 V had to be applied across the memristor. To increase the synaptic weight, 1.5 V was applied to the word line for 150 ns and 0 V was applied to the bit line. Conversely, to decrease the synaptic weight, 0 V was applied to the word line and 1.5 V to the bit line. For the Hebbian training, an M × N memristor ANN was implemented by adopting the single-memristor structure for the M inputs and N outputs, and there were N different classification images. For the guide training, an M × 2N memristor ANN was implemented by adopting the double-synapse memristor structure for the M inputs and N outputs, and there were N different classification images.

### 2.3. Hebbian Training Method

To train the memristor neural network in an unsupervised learning manner, we used the Hebbian training method shown in Table 2. The synaptic connections between input data without output increased. On the contrary, the synaptic connections between output data without input were decreased. Otherwise, the synaptic weights remained the same. In the table, while 1 represents the existence of input or output, 0 represents the absence of input or output.

### 2.4. Guide Training Method

The guide-training algorithm literally guides the memristor neural networks to make them perform a cognitive task, and it utilizes the features of both the Hebbian algorithm and a supervised learning algorithm. The Hebbian learning algorithm updates the synaptic weights only according to the correlations between the inputs and outputs. Thus, there are no mathematical formulas or computations to deduce the change in the synaptic weight. One of the significant drawbacks of unsupervised learning is that the learning results are unpredictable. Training results can differ with different initial synaptic weights even if the training data are the same. In contrast, supervised learning algorithms are based on mathematical formulas. Synaptic weights are changed according to these formulas so that the neural network can respond as the supervisor intended. However, the mathematical computations are very complex. The guide-training algorithm proposed in this paper updates synaptic weights according to the correlation information between the input, output, and intended target output determined by the supervisor. It guides the synaptic weights with this information so that the neural network can respond according to the predefined learning pattern. The guide-training algorithm does not compute derivations or integrations as the backpropagation algorithm does. It just compares the correlations between the inputs and the outputs and then determines whether the synaptic weights increase or decrease. This extremely simple learning algorithm is highly suitable for implementing and training nonlinear memristor neural networks. 

A double-synapse structure was used for the guide training with two synapses for a single pair of input and output. For the M inputs and N different target classification images, an M × 2N double-synapse memristor array was constructed. M inputs were applied to the rows, and the {2 × j − 1}th column and the {2 × j}th column were the positive column (PCj) and the negative column (NCj) of output neuron j (Nj) for every N output neuron. The specific guide training method used in this paper is shown in Table 3. While 1 represents the existence of input or output, 0 represents the absence of input or output. K represents the type of input data, and T represents the predefined target output neuron for this input data. Users can define this learning pattern. In this study, only the input data and predefined training pattern were considered. Only the synaptic weights where input existed were updated. For instance, if the target output neuron for the K input image was T, and the i-th input existed, then the positive-column synaptic weight of the target output neuron increased. The negative-column synaptic weight of the target output neuron decreased. The positive-column synaptic weights of the other non-target output neurons decreased, and the negative-column synaptic weights of the other non-target output neurons increased.

### 2.5. Training and Inference Dataset

**Figure 3:**
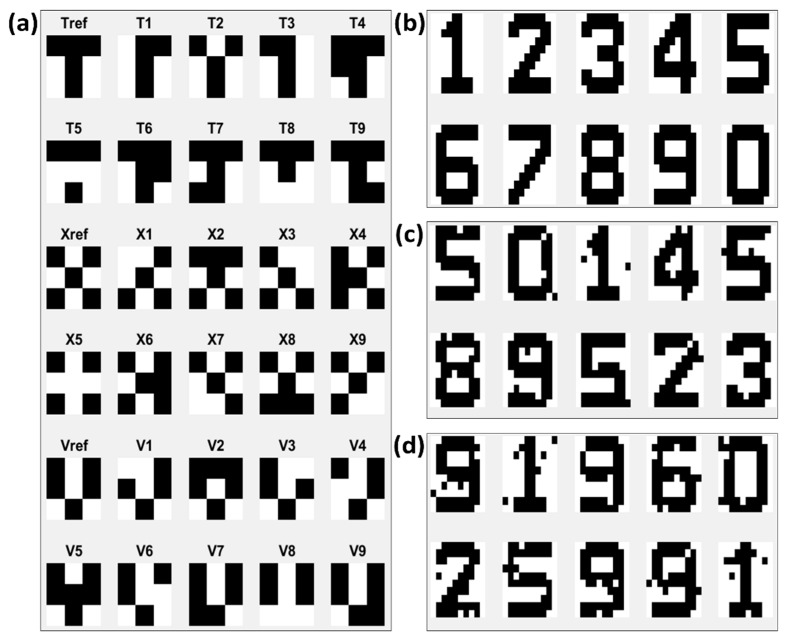
(**a**) 3 × 3 T, X, and V letter images. Tref, Xref, and Vref are original letter images. T1 to T9, X1 to X9, and V1 to V9 are one-pixel flipped noise images of Tref, Xref, and Vref; (**b**) 10 × 10 digit images; (**c**) 3% noise image data of 10 × 10 digit images (three randomly chosen pixels among 100 pixels are flipped); and (**d**) 5% noise image data of 10 × 10 digit images (five randomly chosen pixels among 100 pixels are flipped).

For every new training trial, the memristor ANN was randomized before training. To train the 3 × 3 T, X, and V letter images (corresponding to Tref, Xref, and Vref in Figure 3a), 135 images were contained in a single training dataset: 45 images of each Tref, Xref, and Vref images. The arrangements of T, X, and V images in a single training dataset were randomized. Thus, if 30 sets of training data were used for a single learning trial, then the arrangements of T, X, and V images in all 30 datasets were different. The original image data and one-pixel flipped images (Figure 3a) of the original image data were used to perform the inference simulations. 

To make the memristor ANN learn the 10 × 10 digit images (Figure 3b), 2,708 of the original digit images were used for the training. Three different levels of inference tests were conducted: noise 0% images, noise 3% images, and noise 5% images. These images are shown in Figure 3b–d, respectively. The noise 3% images consisted of images with three randomly chosen pixels flipped. For each digit, 50 different noise images were tested.

## 3. Results

### 3.1. Inference Results after Hebbian Training

**Figure 4:**
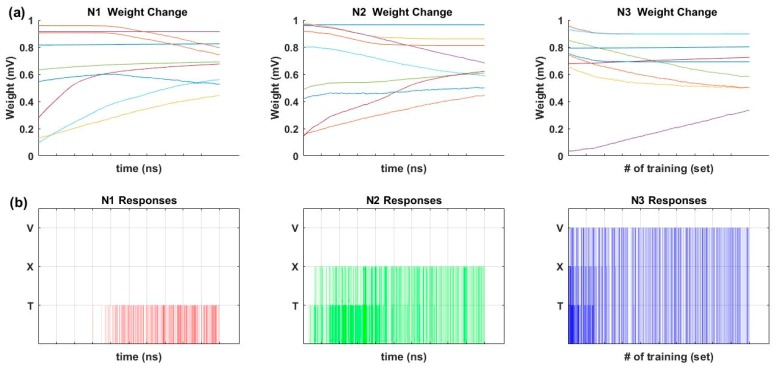
(**a**) Synaptic weight changes during Hebbian training; and (**b**) output neuron responses during Hebbian training.

Synaptic weights were trained according to the Hebbian training method shown in Table 2. Figure 4a shows the changing pattern of synaptic weights during the Hebbian training. Figure 4b shows the output responses of each output neuron during the Hebbian training. For the initial stage of training, output neuron 1 (N1) did not respond to any input image, output neuron 2 (N2) responded to both T and X images, and output neuron 3 (N3) responded to T, X, and V images. However, as the training continued, N1 trained to the T image, N2 trained to the X image, and N3 trained to the V image. The Tref, Xref, Vref, T1, T3, X1, X3, V1, and V3 images in Figure 3a were used for the inference test after the Hebbian training. Table 4 shows the initial voltages of the memristor ANN used for the training in Figure 4. The memristor ANN was randomized before every new training. The average accuracy of the inference test of Tref, T1, T3, Xref, X1, X3, Vref, V1, and V3 was 100%, 97.62%, 100%, 100%, 95.24%, 97.62%, 100%, 95.24%, and 90.48%, respectively.

### 3.2. Inference Results after Guide Training

#### 3.2.1. Inference Results of 9 × 6 Memristor Neural Network 

**Figure 5:**
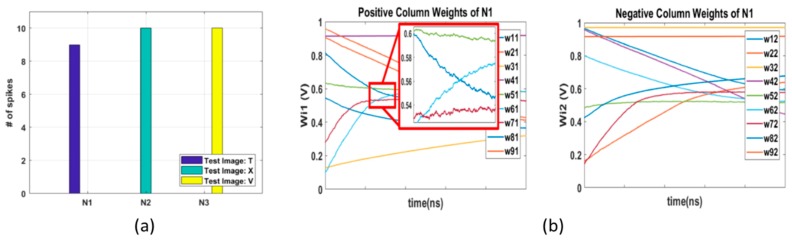
(**a**) 3 × 3 T, X, and V letter image classification test results. Nine different T images were responded to by N1, 10 different X images were responded to by N2, and 10 different V images were responded to by N3. The test results show that the memristor ANN was successfully trained as the predefined learning pattern; (**b**) synaptic weight changes of output neuron 1 during 50 sets of guide training. wij represents the memristor conductance between the i-th top electrode and j-th bottom electrode.

Output neuron 1 was targeted to learn the T image, output neuron 2 was targeted for the X image, and output neuron 3 was targeted for the V image. The inference test was performed after the 50 sets of guide training with this predefined training pattern. For the inference test, the 30 test images in Figure 3a were used. In the best result case, 10 different T images were responded to by output neuron 1 (N1), 10 different X images were responded to by N2, and 10 different V images were responded to by N3. The test results were the same as the predefined learning pattern, and the error rate was zero. Figure 5a shows the inference test results with error after the 50 sets of guide training. Nine different T images were responded to by N1, and the other images of letters X and V were responded to by N2 and N3, respectively. Thus, the single non-responding case of N1 to a T test image was counted as an error. The average accuracy of T, X, and V letter image classification was 92%, 99%, and 100%, respectively. The changes in the 18 synaptic weights of output neuron 1 are shown in Figure 5b. 

#### 3.2.2. Inference Results of 100 × 20 Memristor Neural Network

**Figure 6:**
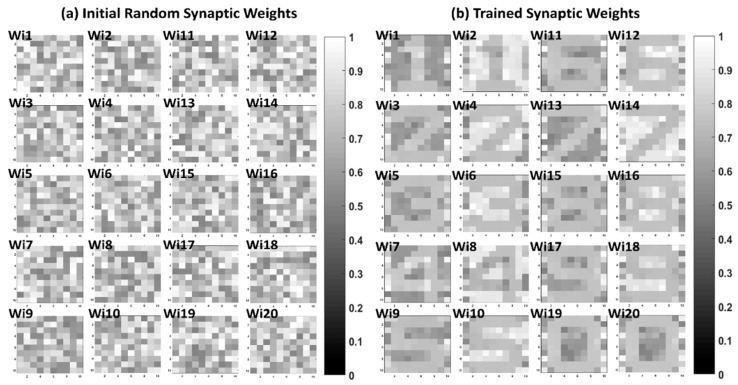
Synaptic weight matrix before and after guide training. A 100 × 20 memristor neural network is utilized for 10 × 10 digit image classification. Each output neuron has positive weights and negative weights. Wi1 represents the positive-column weights of output neuron 1, and Wi2 represents the negative-column weights of output neuron 1. The 100 memristor synapses of 20 columns are shown in the 10 × 10 2D images. (**a**) Initial random synaptic weights. (**b**) Trained synaptic weights after guide training. Trained synaptic weights are trained according to the predefined learning pattern.

In order to train digit images (Figure 3b), 2,708 of the original digit images were used for the training. For the 10 × 10 digit image classification, the learning pattern was predefined as follows: digit 1 was set to output neuron 1, digit 2 was set to output neuron 2, …, digit 9 was set to output neuron 9, and digit 0 was set to output neuron 10. Thus, we expected the corresponding output of the digit 0 image to be [0, 0, 0, 0, 0, 0, 0, 0, 0, 1]. Figure 6a shows the initial random synaptic weights before the guide training, while Figure 6b shows the trained synaptic weights after the guide training. As shown in Figure 6b, the positive and negative synaptic weights of output neuron 1, Wi1 and Wi2, were successfully trained in the shape of digit 1. Other output neurons were also trained as intended. The average accuracy of the inference test of each noise image in Figure 3b–d is shown in Table 5.

## 4. Discussion

In a real on-chip simulation, training has to be conducted with random, nonlinear memristor arrays. In this study, training was conducted on a random memristor array without any initialization process, considering the real-world applications. Unsupervised learning with the Hebbian training method was performed using the proposed neuromorphic hardware system with a nonlinear random memristor ANN, and it successfully classified images. In addition, a new training algorithm optimized to train memristor neural networks was developed. The guide-training algorithm only uses the correlations between the inputs and the outputs like the Hebbian learning algorithm, but the supervisor can configure the training pattern. The training of memristor neural networks poses many intrinsic problems related to the device characteristics. In contrast, the guide-training algorithm proposed in this paper is sufficiently simple to be implemented in an actual circuit and is effective enough to train a memristor neural network. With the guide training algorithm, the 3 × 3 T, X, and V letter image classification and the 10 × 10 digit image classification were successfully conducted with the nonlinear random memristor neural network. The proposed neuromorphic hardware system and guide training algorithm have the potential to train more enhanced memristor ANNs. In the 10 × 10 digit image classification, the digits with large common sections were responded to by corresponding output neurons. The flipped images of digits 5, 6, and 8 were usually responded to by N5, N6, and N8. Moreover, the flipped images of digits 2 and 7 were usually responded to by N2 and N7. Thus, the untrained synapses, which corresponded to the background images, are considered the main contributor to those unintended inference responses. Ongoing studies on the different approaches of the guide-training algorithm are being conducted to overcome these background effects. 

## Figures and Tables

**Table 1:** Memristor device modeling parameters.

Symbol	Value	Symbol	Value
a_1_	0.05	A_n_	6 × 10^3^
a_2_	0.05	x_p_	0.5
b	0.05	x_n_	0.5
V_p_	0.75 V	α_p_	10
V_n_	0.75 V	α_n_	10
A_p_	6 × 10^3^	x_o_	0.5

**Table 2:** Hebbian training method.

Input	Output	Modification
1	1	Remained
1	0	Increased
0	1	Decreased
0	0	Remained

**Table 3:** Guide training method.

Input Image	Predefined Output Neuron	i-th Input	W (i, 2 × j − 1) j = T	W (i, 2 × j) j = T	W (i, 2 × j − 1) j ≠ T	W (i, 2 × j) j ≠ T
K	T	1	Increased	Decreased	Decreased	Increased
		0	Remained	Remained	Remained	Remained

**Table 4:** Initial random weight W (i, j) (mV).

i	W (i, 1)	W (i, 2)	W (i, 3)
1	814.7	964.8	792.0
2	905.7	157.6	959.4
3	126.9	970.5	655.7
4	913.3	957.1	35.7
5	632.3	485.3	849.1
6	97.5	800.2	933.9
7	278.4	141.8	678.7
8	546.8	421.7	757.7
9	957.5	915.7	743.1

**Table 5:** Average accuracy of inference test of 10 × 10 digit image classification.

Noise %	Digit 0	Digit 1	Digit 2	Digit 3	Digit 4	Digit 5	Digit 6	Digit 7	Digit 8	Digit 9
0	100%	100%	100%	100%	100%	98%	100%	96%	100%	100%
3	100%	100%	97%	96%	100%	91%	95%	100%	84%	100%
5	99%	100%	95%	93%	100%	84%	88%	86%	84%	92%
